# Increased Cytokines Response in Patients with Tuberculosis Complicated with Chronic Obstructive Pulmonary Disease

**DOI:** 10.1371/journal.pone.0062385

**Published:** 2013-04-23

**Authors:** Shenjie Tang, Haiyan Cui, Lan Yao, Xiaohui Hao, Yun Shen, Lin Fan, Hua Sun, Zhanjun Zhang, Jian An Huang

**Affiliations:** 1 Department of Respiratory Medicine, The First Affiliated Hospital of Soochow University, Suzhou, China; 2 Tuberculosis Center for Diagnosis and Treatment, Shanghai Pulmonary Hospital, Tongji University School of Medicine, Shanghai, China; University of California, Riverside, United States of America

## Abstract

**Objectives:**

To explore the change and its significance of cytokines in patients with pulmonary tuberculosis complicated with COPD.

**Methods:**

The immune function of 152 cases of pulmonary tuberculosis with COPD was detected to compare with 150 cases of patients with pulmonary tuberculosis, 157 cases of patients with COPD and 50 cases of healthy volunteers who were in the hospital during the same period. T lymphocyte cell population in peripheral blood was detected by flow cytometry. The serum levels of sIL-2R, IL-6, IFN-γ, TNF-α were measured using ELISA.

**Results:**

The percentage of CD4+ T cells in TB patients with or without COPD and COPD patients without TB was significantly lower than that in control group. The percentage of CD4+ T cells in patients with TB and COPD was significantly lower than that in the non-COPD TB patients. The percentage of CD8+ T cells was higher in the TB patients group than that in control group. The CD4+/CD8+ ratio in the TB patients group was significantly lower than that in control group. The concentrations of sIL-2R, IL-6, TNF-α, IFN-γ in TB patients with or without COPD and COPD patients without TB were significantly higher than those in control group. In addition, sIL-2R, IL-6, TNF-α concentrations in the patients with TB and COPD were higher than those in the non-COPD TB patients. The concentrations of sIL-2R, IL-6, TNF-α, IFN-γ in COPD patients with TB were significantly higher than those in COPD patients without TB. There was a significant negative correlation between serum levels of TNF-α, IL-6 and FEV1 (%, predicted) in COPD without TB group.

**Conclusions:**

The patients with pulmonary tuberculosis complicated with COPD were impaired in cellular immunity, and its extent of immune impairment is more serious than those of the patients with pulmonary tuberculosis and the patients with COPD.

## Introduction

In recent years, pulmonary tuberculosis (TB) has begun to rebound quickly worldwide, especially in developing countries. China is one of the world’s 22 countries with the highest burden of TB [1]. Meanwhile, the prevalence of Chronic Obstructive Pulmonary Disease (COPD) is increasing worldwide. It is estimated that COPD will become the third-leading cause of death by 2020 [2]. Previous studies have shown that COPD is a frequent comorbid condition in TB patients. Different studies show that COPD is the one of independent risk factors for death in TB patients [3] and associated with drug-resistant TB [4].These results have raised concern that the increasing global burden of COPD will further enhance the incidence of active TB especially in settings with high burden of TB.

It has been reported much that TB outcome and/or evolution partly depends on the manipulate host immunity by inducing a complex array cytokines [5], [6]. However, the immune responses from TB complicated with COPD patients were rarely studied. These responses can be characterized by measuring the lymphocyte populations and cytokines in circulating in TB with COPD patients.

Therefore, in the present study, in order to evaluate immune responses, the authors investigated the percentages of CD4+ T-lymphocytes, CD8+ T-lymphocytes and the ratio of CD4+ and CD8+ T-lymphocytes in peripheral blood, and further evaluated concentrations of interferon-γ, soluble interleukin-2 receptor,interleukin-6,tumor necrosis factor-α in serum from TB patients with or without COPD and healthy volunteers. To our knowledge, this might be the first study to evaluate immune profiles in TB complicated with COPD patients.

## Patients and Methods

### Patients and Inclusion Criteria

Patients newly diagnosed with pulmonary TB, with or without COPD were randomly selected from patients hospitalized of Shanghai Pulmonary Hospital, Shanghai, China, from January, between January 2009 and March 2012. The diagnosis of pulmonary TB was based on clinical presentation and chest computed tomography (CT) examination, and confirmed by *M.* tuberculosis-positive sputum culture.

In our study, COPD in all the patients with TB and COPD had been diagnosed before TB was diagnosed. COPD was diagnosed according to diagnostic criteria from the guidelines of GOLD Executive Summary [7]. It also presented airflow limitation on spirometry (FEV1/forced vital capacity <70%), as defined by the Global Initiative for Chronic Obstructive Lung Disease initiative [7]. Patients with COPD who had mild to moderate airflow obstruction were enrolled. They were clinically stable and had not had an exacerbation episode during the past three months.

Age- and sex- matched healthy control non-smoking subjects with normal chest radiographic were selected from a population who had undergone an annual health check-up.

Subjects in the non-TB groups were screened for active TB by chest X-ray. All subjects with signs and symptoms suggesting active TB, with a history of prior of anti-TB treatment were excluded from the study. All patients performed a spirometry. All patients and healthy volunteers were negative for human immunodeficiency virus and did not have other systemic autoimmune disorders or history of immune-suppressive therapy. Written informed consent was obtained from all participants. The study was approved by the Ethics Committee of Shanghai Pulmonary Hospital.

### Sample Collection and Assessment

Blood samples from patients and healthy controls were drawn before therapy. Peripheral blood mononuclear cells (PBMC) were isolated by Ficoll-Hypaque gradient centrifugation. T lymphocyte cells population were detected by flow cytometry double-labeled antibody. Flow cytometry analysis was performed using the Beckman coulter Cytomics FCS500 flow cytometry system (Beckman Coulter) according to the manufacturer’s instructions. Data were analyzed using CXP Analysis software (Beckman).

The cytokines were measured by commercially available ELISA kits (Shanghai Senxiong Biotech Company) according to the manufacturer’s instructions. The enzyme-linked immunosorbent assay (ELISA) was performed in duplicate for each sample and the cytokine concentrations were calculated using standard curves.

### Statistical Analysis

The analysis was performed using the nonparametric Kruskal-Wallis test to compare the immune parameters of TB with COPD patients, TB without COPD patients and healthy individuals, followed by the Mann-Whitney U test to compare two TB groups. Correlations between different parameters were determined by spearman’s rank correlation coefficient. The analysis was performed using the statistical software SPSS 15.0 for Windows (SPSS Inc., IL). *P* values *<*0.05 were considered significant.

## Results

The study population consisted of 152 TB with COPD patients, 150 TB without COPD patients, 157 COPD without TB and 50 healthy volunteers. Baseline characteristics of the four groups are presented in table 1. There was no age and sex ratio difference among groups (*P*>0.05). The number of smokers in TB patients with COPD and COPD patients without TB was significantly more than that in the non-COPD TB patients (*P<*0.05).

**Table 1:** Characteristics of four groups.

	TB with COPD (n = 152)	TB without COPD (n = 150)	COPD without TB (n = 157)	Healthy control (n = 50)
	N/mean (% or SD)	N/mean (% or SD)	N/mean (% or SD)	N/mean (% or SD)
Age	59.73(11.41)	48.13(15.67)	60.17(9.41)	54.32(19.51)
Male	104(68.42)	105(70)	117(74.52)	34(68)
female	48(31.58)	45(30)	40(25.48)	16(32)
smoking				
Never-smoker	45(29.61)	78(52)	48(30.57)	50(100)
Former smoker	65(42.76)	52(34.67)	62(39.49)	0
Current smoker	42(27.63)	20(13.33)	47(29.94)	0
FEV1(%, predicted)	72.91(10.37)	94.57(4.91)	75.14(10.53)	98.22(3.79)
With comorbidity				
diabetes	7(4.6)	9(6)	7(4.46)	0
Cardiac disease	12(7.89)	9(6)	13(8.28)	0
liver disease	6(3.95)	7(4.67)	6(3.82)	0

### Comparison of the Percentages of CD4+ T Cells and CD8+ T Cells, and CD4/CD8 Ratio

**Figure 1:**
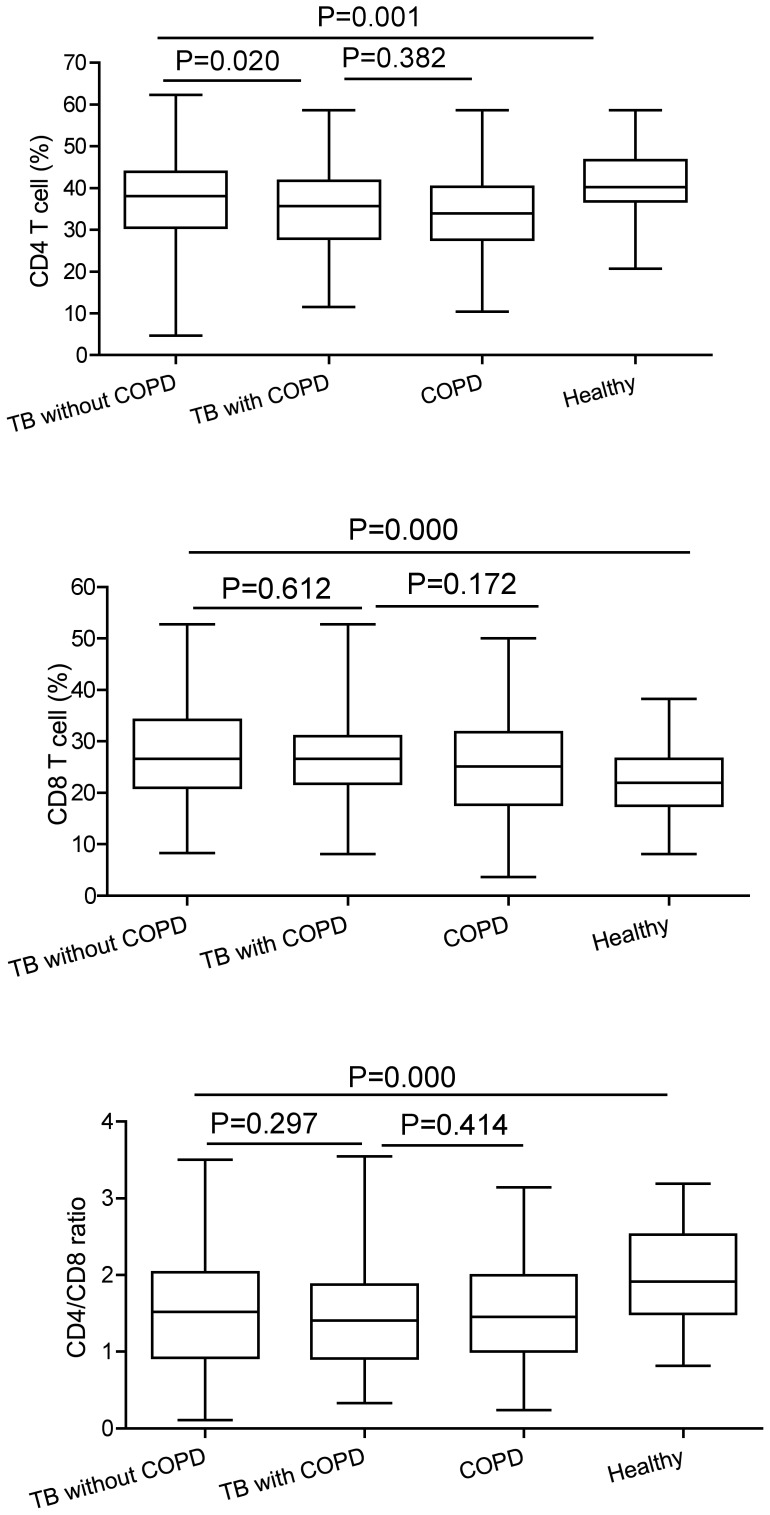
Comparison of the percentages of CD4,CD8 and CD4/CD8. Serum percentages of CD4+ T cells, CD8+ T cells and the CD4/CD8 ratio in TB patients, TB with COPD patients, COPD patients and healthy controls. Horizontal bars represent median values, boxes represent the interquartile range (25–75%) and whiskers represent the highest and the lowest values. Horizontal lines indicate a statistically significant difference between groups.

The data in Fig. 1 show the percentage of CD4+ T cells, CD8+ T cells, and the CD4+/CD8+ ratio in the sera of patients and healthy controls. The percentage of CD4+ T cells in TB patients with or without COPD and COPD patients without TB was significantly lower than that in control group (*P*≤0.001). The percentage of CD4+ T cells in patients with TB and COPD was significantly lower than that in the non-COPD TB patients (*P* = 0.020). The percentage of CD8+ T cells was higher in the TB patients group than that in control group (*P* = 0.000). The CD4+/CD8+ ratio in the TB patients group was significantly lower than that in control group (*P* = 0.000). There was no significant difference of the percentage of CD8+ T cells and the CD4+/CD8+ ratio between the two TB groups. There was no significant difference of the percentage of CD4+ T cells, CD8+ T cells and the CD4+/CD8+ ratio between the two COPD groups.

### Comparison of Cytokine Levels between the TB and Control Groups

**Figure 2:**
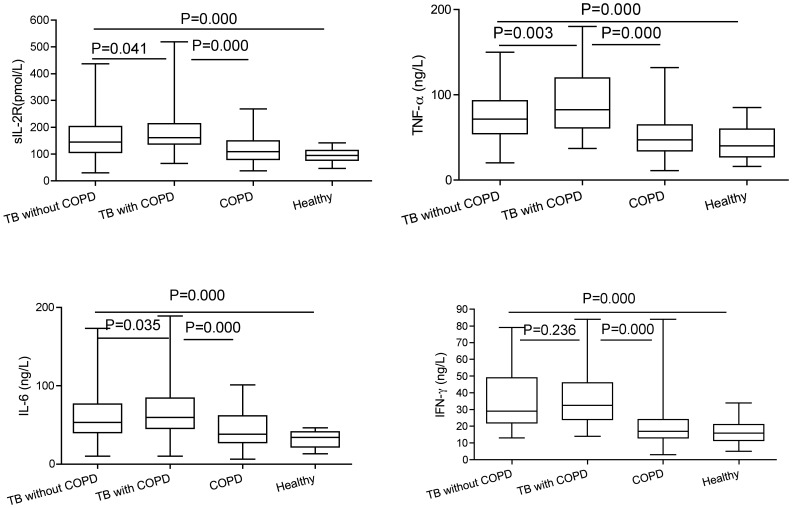
Serum levels of cytokines in different group. Serum levels of IL-2R, TNF-α, IL-6 and IFN- γ in TB patients, TB with COPD patients, COPD patients and healthy controls. Horizontal bars represent median values, boxes represent the interquartile range (25–75%) and whiskers represent the highest and the lowest values. Horizontal lines indicate a statistically significant difference between groups.

The differences in the levels of four cytokines in serum among the four groups are shown in Figure 2. The levels of sIL-2R, IL-6, TNF-α, IFN-γ in TB patients with or without COPD were significantly higher than those in control group and COPD patients without TB (*P* = 0.000). In addition, sIL-2R, IL-6, TNF-α levels in the patients with TB and COPD were higher than those in the non-COPD TB patients (*P* = 0.041, *P* = 0.003, *P* = 0.035, respectively). The levels of sIL-2R, IL-6, TNF-α, IFN-γ in COPD patients with TB were significantly higher than those in COPD patients without TB (*P* = 0.000).

### Serum Cytokine Levels and Pulmonary TB Severity

**Figure 3:**
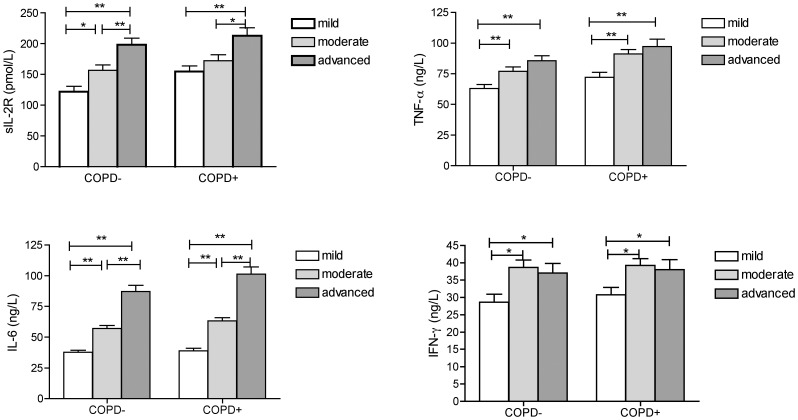
Serum cytokines level in tuberculosis patients with different degree of pulmonary involvement. Bars indicate the mean ±SEM for each group. Horizontal lines indicate a statistically significant difference between groups. (*P<0.05, **P<0.01).

According to the extent and type of chest radiograph findings, the severity of pulmonary TB was classified into three groups: mild (a single lobe involved, and without visible cavities, without COPD n = 38, with COPD n = 34), moderate (unilateral involvement of two or more lobes and cavities, if present, reaching a total diameter no greater than 4 cm, without COPD n = 63, with COPD n = 74), and advanced (massive involvement of both lungs and multiple cavities, without COPD n = 49, with COPD n = 44). Data in Fig. 3 show that serum levels of IL-6, TNF-α, IFN-γ in patients with moderate and advanced TB were significantly increased as compared to mild TB; serum sIL-2R, IL-6 levels of patients with advanced TB were significant higher than those of patients with mild or moderate TB.

### Correlation between T Cell Frequencies and Levels of Each of the Cytokines and Cytokine Receptor and FEV1 of Predicted in COPD Patients

**Figure 4:**
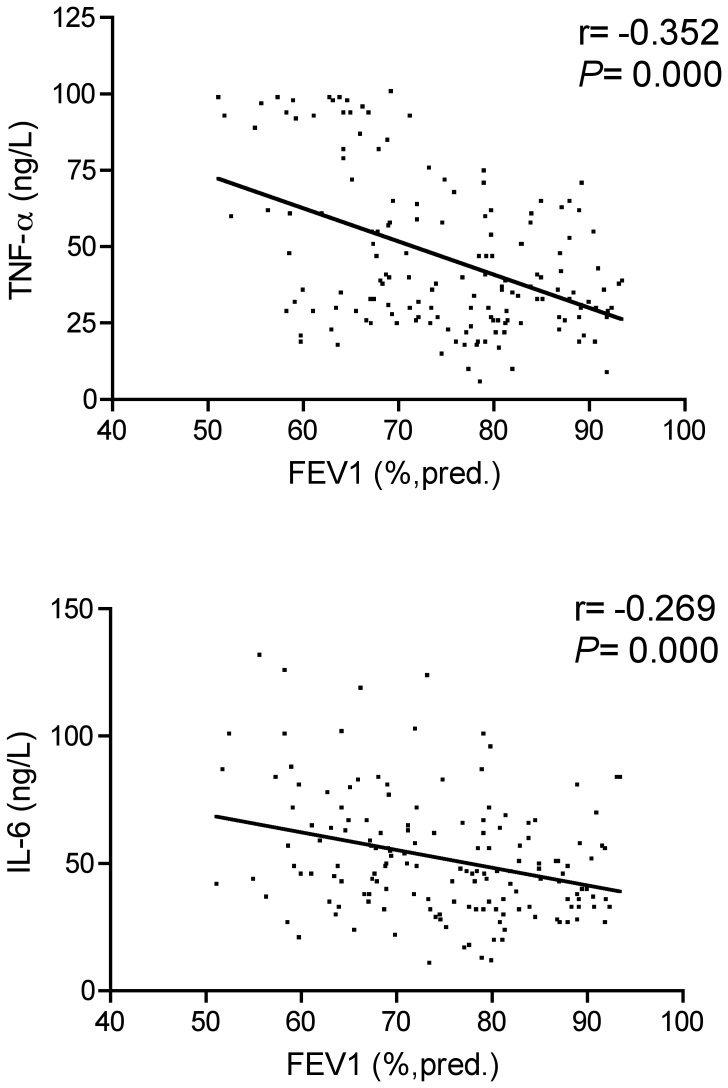
Correlations between TNF-a, IL-6 and FEV1 in COPD without TB patients.

Of measured immune parameters, only TNF-α and IL-6 showed negative significant correlations with FEV1 of predicted in patients with mild-moderate COPD without TB (*P* = 0.000) (Figure 4). There was not, however, a relationship between FEV1 of predicted and serum levels of cytokines and cytokine receptor either in TB patients or TB with COPD patients (data not shown).

## Discussion

T lymphocytes, especially CD4+ T lymphocytes, play an important role in the immune reaction against TB. Indeed there are many inter- and intracellular processes involved; and if some of these mechanisms can be elucidated, new treatment strategies may be developed. Although a Th1 profile is necessary for a protective response, it may also cause immunopathologic damage. Therefore, a Th2 response might have important regulatory effects on protecting patients from collateral host tissue damage. The classical cytokines such as IL-2, TNF-α, IL-6 and IFN-γ, have been reported to correlate with disease activity during active pulmonary TB [8].

There is no doubt about the essential protective role of CD4+ T cells in controlling tuberculosis infection. Stimulating CD4+ T-cell can produce Th1-associated cytokines, which may increase Th1 response. It has been demonstrated that CD8+ T cells are also necessary for immunity against tuberculosis in a variety of animal models as well as in humans [9], [10]. Our study showed that the immune profile in the TB with COPD patients was different from that in the TB without COPD patients and healthy controls. The percentage of CD4+ T cells in TB patients with or without COPD was significantly lower than that in control group (*P*≤0.001). The percentage of CD4+ T cells in patients with TB and COPD was significantly lower than that in the non-COPD TB patients (*P* = 0.020). The percentage of CD8+ T cells was higher in the TB patients group than that in control group (*P* = 0.000). The CD4+/CD8+ ratio in the TB patients group was significantly lower than that in control group (*P* = 0.000). On the other hand, some studies indicate that CD3+ and CD4+ cell counts may be higher in drug-sensitive pulmonary TB cases [11]. Koch et al [12] also reported that peripheral blood T-lymphocyte abnormalities might be involved in the pathogenesis of airflow limitation. The results showed that patients with TB had cellular immune damage. In addition, the extent of cellular immune impairment in patients with pulmonary tuberculosis complicated by chronic obstructive pulmonary disease was more serious than that of the patients with pulmonary tuberculosis. Chronic obstructive pulmonary disease (COPD) is associated with pulmonary and systemic inflammation. COPD has a significantly higher proportion of CD8+ T lymphocytes and a lower CD4+/CD8+ ratio than that in the healthy smokers [13]. We also found that the percentage of CD4+ T cells in COPD patients without TB was significantly lower than that in control group (P≤0.001). However, there was no significant difference of the percentage of CD4+ T cells, CD8+ T cells and the CD4+/CD8+ ratio between the two COPD groups. Paats et al found significantly increased proportions of IFNγ+ and TNFα+CD8+ T cells in COPD patients, when compared with healthy controls. In contrast, expression profiles in circulating CD4+ T cells were similar in COPD patients and healthy controls for all cytokines tested [14]. Both CD4+ and CD8+ T lymphocytes play a key role in COPD pathogenesis.

It has been reported that cytokines are participated in the response and immune reaction of TB [15], [16]. Th1 cells secrete IL-2, IFN- γ and other cytokines to participate in cellular immunity. Study showed that proinflammatory cytokines, TNF-α and IL-6 also are related to pathologic process [17]. When COPD patients’ respiratory tract is infected by viruses or bacteria, some antigen components or metabolites in viruses or bacteria such as LPS, endotoxin can activate alveolar macrophages to produce TNF-α, IL-2 and other inflammatory factors, and these inflammatory factors in turn can promote alveolar macrophages or bronchial epithelial cells to produce IL-6. Cytokines produced by local tissue are absorbed into the bloodstream and endotoxin causes monocyte-macrophage cells to be activated, thus resulting in a rise of blood TNF-α, IL-6 [18], [19]. Multiple cytokines play a role in the orchestration of inflammation in chronic obstructive pulmonary disease, through the recruitment, activation and survival of inflammatory cells [20]. Studies have shown that whether in acute exacerbation of COPD or after treatment, the levels of IL-6 and TNF-α are higher than those of the healthy control group. This suggests that COPD patients have an excessive production of lL-6 and TNF-α [21], [22].

The soluble interleukin-2 receptor (sIL-2R) is released along with interleukin-2 from activated T lymphocytes. The main function of sIL-2R is regulation of the immune response by binding IL-2, which results in blocking the biological functions of this cytokine. By the competition with cell surface IL-2 receptor, sIL-2R acts as immunosuppressive factor inhibiting IL-2R-realated lymphoblast growth [18], [19]. sIL-2R might be a valuable marker for forecasting and monitoring TB outcome before and after chemotherapy [23]. In this study, the levels of sIL-2R in TB patients with or without COPD were significantly higher than those in control group (*P* = 0.000), and the levels of sIL-2R in the patients with TB and COPD were higher than those in the non-COPD TB patients (*P* = 0.041). Meanwhile, the levels of sIL-2R in COPD patients with TB were significantly higher than those in COPD patients without TB (*P* = 0.000). This is likely to reflect a severe immune compromised in TB with COPD patients.

Interleukin-6 (IL-6) is proinflammatory cytokine that produces multifunctional effects. It is important in the pro-inflammatory response and has recently been shown to be one of the most important biomarkers in TB [24]. IL-6 as a proinflammatory cytokine may play a considerable role in the systemic inflammatory response in COPD. It has been shown that high levels of serum IL-6 are associated with impaired lung function or a faster decline in lung function [25]–[27]. In addition, elevated serum levels of IL-6 have been associated with dyspnea, skeletal muscle weakness, pulmonary arterial hypertension, and COPD exacerbations [28]. Djoba et al [29] has reported pro-inflammatory cytokines such as IL-6 are increased in subjects with active TB disease compared to those with latent tuberculosis infection (LTBI). In the present study, we found that the level of IL-6, TNF-α, IFN-γ in TB patients with or without COPD and COPD patients without TB was significantly higher than those in the healthy controls. In addition, IL-6 level in the patients with TB and COPD was higher than those in the non-COPD TB patients and the COPD patients without TB. We reasoned that increased IL-6 in TB complicated COPD might be related to the induction and maintenance of inflammatory response. However, whether it plays a protective role in immune, or immune injury, or a double-edged sword still need further study.

TNF-α mediates early inflammatory response against pathogens and is produced by a variety of cells including macrophages, lymphocytes, neutrophils, mast cells, and endothelial cells [30]. It is considered necessary to remove bacteria in inflammatory lesions. It has been found that TNF-a is important in the control of *Mtb.* infection [24], [31]. Higher TNF-α level in TB with COPD patients as compared to the other three groups were found here. Sutherland et al [32] found that TNF-a production were significantly higher in active TB. However, the role of TNF-α has also been postulated to be dose dependent with too high levels associated with increased TB pathology [33]. High levels of the cytokine may induce an excessive inflammatory response, tissue necrosis and cachexia or wasting. Moermans et al [34] also demonstrated that overproduction of TNF-α at systemic level correlates with disease severity. These data suggested TNF-α may be involved in the pathogenesis of pulmonary tuberculosis complicated by COPD.

INF-γ is an important Th1 cytokine produced by CD4+ and CD8+ T cells. It strongly promotes the T helper 1 (Th1) cell response. Moreover, it has been demonstrated to act as a protective factor against tuberculosis in human and animal studies [35], [36]. INF-γ participates in the pathogenesis of inflammation in chronic obstructive pulmonary disease [37]. A significantly increased production of IFN-γ has been observed in PTB [8], [38]. In this study, we found that the level of IFN-γ in COPD patients with TB were significantly higher than those in COPD patients without TB and in the healthy controls. However, we did not observe a significant difference between the TB with COPD patients and TB without COPD patients. This is likely to reflect that the protective immune is not increase in TB with COPD patients.

Previous studies have also suggested an association between the levels of cytokines and the severity of tuberculosis [39], [40]. Jurado et al [39] found that IL-17 and IFN-γ expression in lymphocytes from patients with active tuberculosis was correlated directly with clinical parameters associated with disease severity. Another study showed that the levels of sIL-2R and IFN-γ in initial treatment and retreatment cavity pulmonary tuberculosis patients were higher than those in pulmonary tuberculosis patients without cavity [40]. In our study, serum levels of IL-6, TNF-α, IFN-γ in patients with moderate and advanced TB were significantly increased as compared to mild TB; serum sIL-2R, IL-6 levels of patients with advanced TB were significant higher than those of patients with mild or moderate TB. This indicated that there are correlations between the levels of the cytokines and cytokine receptor and the severity of pulmonary TB.

Several studies have suggested TNF-a and IL-6 levels are increased in induced sputum and plasma of patients with COPD, particularly during exacerbations [41], [42]. Soler et al. [43] demonstrated a negative correlation between the FEV1 of predicted and the IL-6 levels in BAL fluid. We also found that there was a significant negative correlation between serum levels of TNF-α and IL-6 and FEV1 (%, predicted) in COPD without TB group. However, there was not a relationship between FEV1 of predicted and serum levels of cytokines and cytokine receptor either in TB patients or TB with COPD patients. This indicates that the levels of IL-6 and TNF-α in serum might be related to the degree of airflow limitation in COPD without TB group. The reason why there is no correlation between FEV1 of predicted and serum IL-6 or TNF-α in TB with COPD group are unknown, which needs further study in the future.

There are several limitations in our study. First, the impact of cigarette smoking has not been studied. Cigarette smoking has been widely recognized as a primary risk factor for the development of COPD and also an important factor affecting the proinflammatory cytokines as an independent factor [44]. However, its impact on the cytokines couldn’t be statistically analyzed in this study as there were no matched numbers of smokers in each group. Further research is needed to elucidate the interaction of these immunological profiles with smoking. Secondly, Airflow limitation might be related to the severity of tuberculosis. However, we have not conducted relevant research, about which we will study in the future. Thirdly, the severity of tuberculosis, such as symptoms, course of disease, sputum bacteriological factor etc. might influence the levels of some cytokines. These need to be further studied in the future.

In conclusion, the present findings have demonstrated that there is CD4+ T lymphocytes depletion in peripheral blood and increased concentrations of sIL-2R, IL-6, TNF-α and IFN-γ in serum in patients with TB complicated by COPD. The patients with pulmonary tuberculosis and the patients with COPD were impaired in cellular immunity. The patients with pulmonary tuberculosis complicated with COPD were also impaired in cellular immunity, and its extent of immune impairment is more serious than those of the patients with pulmonary tuberculosis and the patients with COPD. While IL-6 and TNF-α are essential components of the host protective response against mycobacterial infection, high levels of the cytokine may induce an excessive inflammatory response that overwhelms the beneficial effects of the cytokine. These findings implicated a role for adaptive immunity in the progression of TB with COPD. Further investigation on the immune function in the patients with pulmonary tuberculosis and the patients with COPD will help to clarify the immune pathogenesis and provide the rationale for immune-based interventions.
